# Allergic Mastocytic Gastroenteritis and Colitis: An Unexplained Etiology in Chronic Abdominal Pain and Gastrointestinal Dysmotility

**DOI:** 10.1155/2012/950582

**Published:** 2012-03-12

**Authors:** A. Akhavein M, N. R. Patel, P. K. Muniyappa, S. C. Glover

**Affiliations:** ^1^Division of Gastroenterology, Hepatology, and Nutrition, University of Florida, 1600 SW Archer Road, HD512A, P.O. Box 100214, Gainesville, FL 32610, USA; ^2^Section of Digestive Diseases and Nutrition, Department of Medicine, University of Illinois at Chicago, Chicago, IL 60612, USA; ^3^Department of Medicine, Mercy Medical Center, Chicago, IL 60616, USA; ^4^Department of Pediatrics, University of Illinois at Chicago, Chicago, IL 60612, USA

## Abstract

Abdominal pain, bloating, early satiety, and changes in bowel habits are common presenting symptoms in individuals with functional GI disorders. Emerging data suggests that these symptoms may be associated with mast cell excess and/or mast cell instability in the GI tract. The aim of this retrospective study was to evaluate the contribution of mast cells to the aforementioned symptoms in individuals with a history of atopic disease. A retrospective chart review of individuals seen in a university GI practice was conducted and twenty-four subjects were identified. The majority had abdominal pain, early satiety, and nocturnal awakening. 66.7% and 37.5% had a history of environmental and/or food allergy. Solid gastric emptying was increased as were the mean number of mast cells reported on biopsies from the stomach, small bowel, and colon (>37/hpf) by CD117 staining. Mean whole blood histamine levels were uniformly elevated. This study suggests that in individuals with these characteristics, consideration should be given to staining their gastrointestinal biopsies for mast cells as this may provide them with relatively non-toxic but highly targeted treatment options. Allergic gastroenteritis and colitis may represent a third type of GI mast cell disorder along with mast cell activation syndrome and mastocytic enterocolitis.

## 1. Introduction

Chronic abdominal pain together with symptoms of altered gastrointestinal motility defines a rather common presenting tableau in the gastroenterology practice, shared by idiopathic gastroparesis (IGP) and functional GI disorders such as irritable bowel syndrome (IBS) and functional dyspepsia (FD), among others.

IBS is defined as a chronic continuous or remittent gastrointestinal illness characterized by frequent unexplained symptoms that include abdominal pain, bloating, and bowel disturbance, which may be either diarrhea or constipation or an erratic bowel habit that has features of both. IBS is a common illness with an overall prevalence of about 10–15% within the general population [1–3]. Similar to FD and IGP, IBS also presents with chronic abdominal pain and symptoms of GI dysmotility (GID). Dysmotility is one of the proposed mechanisms in the pathophysiology of IBS [4, 5].

On the other hand, one of the most studied immune cells associated with IBS is the mast cell. Increased numbers of GI mast cells have been documented in a subset of IBS patients throughout the small and large intestine [6]: in the duodenum [7], jejunum [8], ileum [9, 10], cecum [11, 12], ascending and descending colon, and rectum [10]. Mast cells and their mediators play a potential role in the pathophysiology of IBS by causing sensorimotor dysfunction of the gut through interactions with the enteric nervous system [13–15]. Moreover, IBS has been increasingly linked to food allergy [16–19] in which mast cells play a critical role [20].

Another functional GI disorder with chronic abdominal pain and GID symptoms is functional dyspepsia (FD), characterized by abdominal pain and symptoms of GID, such as early satiety and postprandial fullness, without any evidence of structural diseases.

The goal of this retrospective caseseries was to describe a subgroup of patients whose main symptomatology consisted of chronic abdominal pain and symptoms suggestive of GID, who had associated increased numbers of GI mast cells, history of food/environmental allergy, nocturnal awakening, and/or mast cell instability. Gastrointestinal mast cells excess is defined as the presence of greater than 20 mast cells per high-power field of microscopy in the GI tract mucosa. Mast cell instability is marked by an increase in mast cells mediators' release, for example, increased circulating histamine levels.

## 2. Methods

A retrospective chart review of the patients seen at the University of Illinois GI clinics between years 2006 and 2009 was performed. Inclusion criteria were defined as follows:

1. patients with GID symptoms or symptoms of functional disorders such as IBS or FD,
2. patients with available GI tract mucosal biopsies and CD117 staining of the specimens for mast cell evaluation.

The data extracted from the patients' charts included the following:

- demographic data including age, sex, and race,
- history and physical examination with emphasis on GI symptoms: abdominal pain, early satiety, diarrhea, constipation, abdominal bloating, nocturnal awakening, history of food/environmental allergy, history of immunotherapy, and presence of succussion splash,
- laboratory data including CBC, ESR, CRP, serum IgE, whole-blood histamine, and stool studies,
- history of medications, including improvement in GI complaints after being put on medication,
- pathology reports and histological slides,
- radiology reports including nuclear medicine studies and CT scans,
- results of food allergy testing by RAST (radioallergosorbent test) or SPT (skin prick testing),
- CD-25 marker assessment and serum tryptase levels measurement were not recorded since these had already been studied in previous papers.

**Figure 1:**
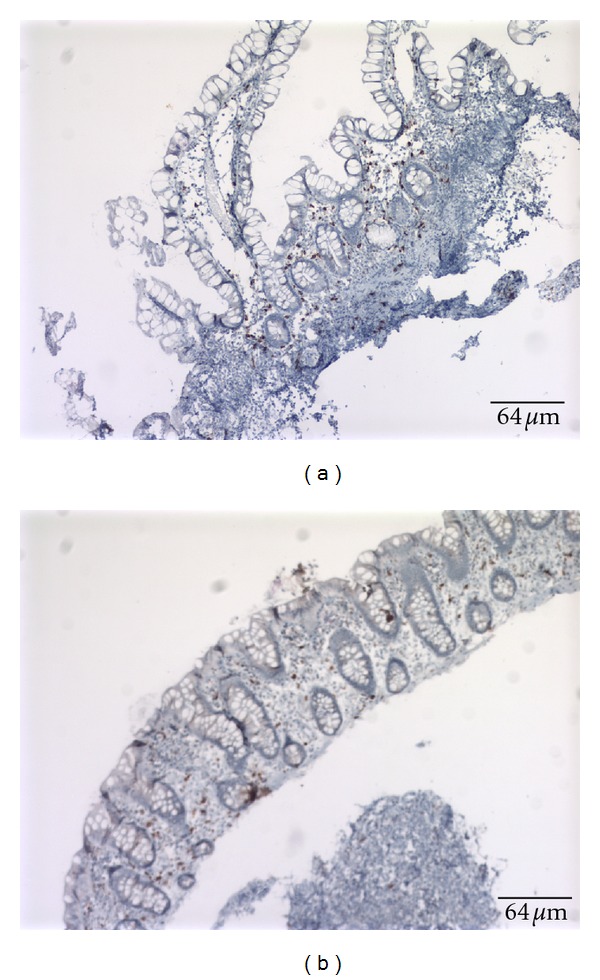
Anti-CD117 staining was used to identify the mast cells. Images of positive CD117 in the small bowel (a) and the colon (b). Positive cells are brown. Abnormal CD117 is considered to be more than 20 cells per high-power field (hpf). Bar length is equal to 64 *μ*m.

Staining options available for mast cells include toluidine blue staining, tryptase staining, the Giemsa staining, and CD117 marker detection [21]. Unlike CD25 which is expressed on neoplastic mast cells [22], the CD117 marker (The *c-Kit* antigen) is detectable both in normal and neoplastic mast cells either by flow cytometry or by immunohistochemistry. The stem cell factor (SCF) receptor *Kit* (CD117) antigen is expressed on all types of mast cells independent of maturation and activation status. Therefore, CD117/*Kit* was used in these patients as a robust mast cell marker antigen (Figure 1) [23]. The biopsies taken were mucosal, so the CD117 (+) cells detected were mucosal mast cells and not interstitial cells of Cajal or submucosal mast cells. CD-25 marker assessment and serum tryptase levels measurement were not done since these had already been studied in previous papers [20, 24].

Delayed gastric emptying was defined as emptying time greater than 200 minutes on solid-phase gastric emptying scan.

The cutoff point for increased number of GI mucosal-mast cells was defined as more than 20 mast cells per high power field (hpf) on microscopy using immunostaining for CD117. Other inflammatory and allergy parameters collected included serum IgE and whole-blood histamine levels. Normal whole-blood histamine level range was defined by our lab as less that 300 nmol/L, which is in concordance with the ranges proposed in the literature [25]. Normal serum IgE level range was defined by our lab as 10–179 IU/mL.

The collected data was analyzed using descriptive statistical methods, with means, standard deviations, and standard errors being reported.

## 3. Results

This population consisted of 21 females and 3 males, with an age range of 16 to 64 and a mean age of 34.5. All the patients had a history of abdominal pain, with 45.8% of them having diarrhea and 37.5% having constipation (Table 1). Of subjects with available data, 5.5% had a history of food allergy only, 50% had a history of environmental allergies only, and 43.7% had both. Specific details of patients' histories and symptoms are summarized in Tables 1 and 2.

**Figure 2:**
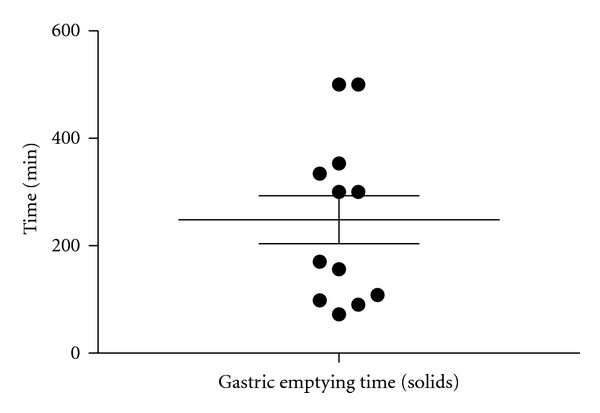
Frequency distribution of solid-phase gastric emptying times. Scan was performed over 90 minutes. Data was available for 14 of 24 patients. Mean emptying time was 204 minutes. Emptying times greater than 200 minutes are suggestive of gastroparesis.

For the 14 out of 24 patients who had a gastric emptying scintigraphy done to explain their upper GI symptoms, the mean emptying time was 204 minutes on the solid-phase gastric emptying scan (Figure 2 and Table 3). The scintiscanning was performed over 90 minutes using the standard protocol at the University of Illinois Nuclear Medicine Division at that time (Figure 2 and Table 3).

**Figure 3:**
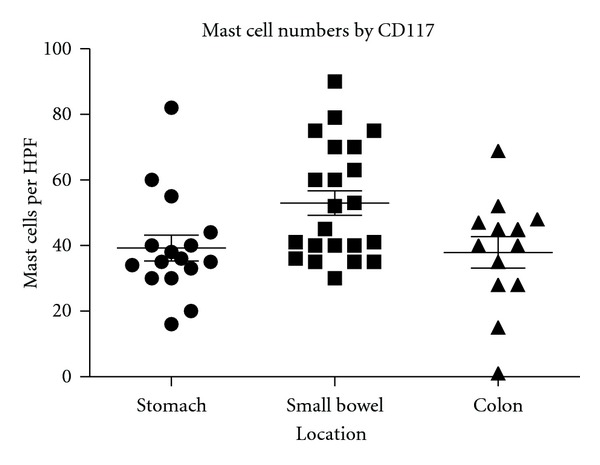
The distribution of mast cells based on the location in the GI tract. Mean number in the stomach was 39 cells per high-power field (hpf). Mean number in the small bowel was 57 cells per hpf. Mean number in the colon was 37 cells per hpf.

The mean number of the mast cells in the stomach was 39 cells per high-power field (hpf), in the small bowel was 57 cells per hpf, and in the colon was 37 cells per hpf (Figure 3 and Table 4). Unlike dense aggregates of mast cells seen in systemic mastocytosis biopsies [26], our specimens showed diffused mast cell infiltration of the mucosa (Figure 1).

**Figure 4:**
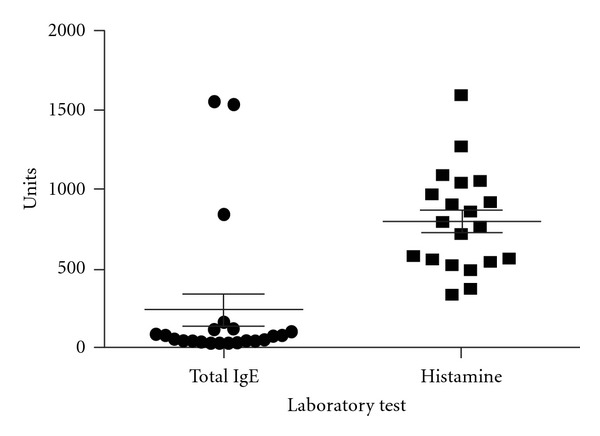
Frequency distribution of serum IgE level and whole blood Histamine levels in the group. Mean IgE level was 213 IU/mL and and Median was 37 IU/mL. Mean Histamine level was 798 nmol/L. Data was available for 22 and 20 patients, respectively.

The inflammatory and allergy markers studied included serum IgE level and whole-blood histamine levels. The mean serum IgE level was 213 IU/mL, and the median was 37 IU/mL. The mean whole-blood histamine level was 798 nmol/L (Figure 4 and Table 5). Elevated serum IgE levels were seen in 13.6% of patients with available data, and 95% of patients with available data had an elevated whole-blood histamine level. Serum IgE levels data was available for 22 patients, and whole-blood histamine levels data was available for 20 patients. Summary of collected values is shown in Tables 3, 4, and 5 and Figures 2, 3, and 4.

## 4. Limitations

The study's retrospective design imposed limitations inherent in the nature of such studies. Therefore, the date presented herein is of a descriptive nature. In spite of this, this current study serves as a basis for future cohort studies and clinical trials that evaluate the role of mast cells in GI disease.

This study uses data from a 90-minute gastric emptying scintigraphy scans. At the time these individuals were seen, the 90-minute gastric emptying study was standard protocol in the University of Illinois Nuclear Medicine division. The authors acknowledge that the 4-hour gastric emptying scan is the new standard of care [27–29].

## 5. Discussion

This paper describes a distinct group of GI patients with chronic abdominal pain and symptoms of GI dysmotility, features that mimic the features of entities such as IBS, FD, or IGP, but who actually suffer from gastrointestinal mast cell excess and/or instability. These patients frequently exhibit features of mast cell excess, including positive history of food and/or environmental allergies, signs and symptoms such as flushing, pruritus, tachycardia, asthma, headache, or dermatographism, and suggestive lab data such as elevated serum IgE levels or whole-blood histamine levels greater than 300 nmol/L [24, 25, 30]. The corresponding plasma histamine level would be 3 nmol/L [25].

In the current literature, there are two loosely defined entities associated with increased numbers on mast cells on gastrointestinal biopsies. The first of these is mastocytic enterocolitis. Mastocytic enterocolitis is defined as more than 20 mast cells per high-power field by tryptase stain in individuals with chronic diarrhea of unknown etiology [20]. Mast cell activation syndrome occurs in individuals who have symptoms associated with mast cell instability including dermatographism, flushing, mental fog, or poor concentration, abdominal pain, diarrhea, anaphylaxis, and asthma who have a dramatic improvement in their symptoms in response to antihistamines and H2 blockers. Intriguingly, in this group, the numbers of mast cells on gastrointestinal biopsies by CD117 or tryptase were between 17 and 23 cells per high-power field [24]. These distinguishing features between our cohort and the two other published GI mast cell disorders are summarized in Table 6.

As mentioned in the introduction to this paper, IBS has been associated with elevated mast cell numbers [6–9, 11, 12] and food allergy [16–19]. The cohort we describe herein has several features that distinguish it from mastocytic enterocolitis and mast cell activation syndrome. They are (1) documented GI dysmotility, (2) nocturnal awakening, (3) elevated histamine levels, (4) history of food or environmental allergy, (5) significantly higher numbers of mast cells per high-power field, that is, approximately 40 per high-power field on average. As such, we propose that our cohort of patients represents a possible third entity wherein elevated numbers of mast cells are noted on gastrointestinal biopsies in patients who have previously been classified as having IBS, functional dyspepsia, or idiopathic gastroparesis. We suggest that this disorder could potentially be referred to as allergic mastocytic gastroenteritis and colitis as these patients have documented allergies, elevated histamine levels, and nocturnal awakening. Nocturnal awakening has been associated with uncontrolled asthma [31] and in our cohort differentiates our patients from those with true IBS as defined by ROME III criteria. We believe that as in asthma, nocturnal awakening in our cohort was due to a spike in leukotrienes.

As other authors have suggested, treatment of gastrointestinal mast cell disease should be focused on blockade of H1 and H2 and mast cell stabilization. These therapeutic approaches include H1 receptor antagonists such as diphenhydramine (Benadryl), cetirizine (Zyrtec), loratadine (Claritin) [13], H2 receptor antagonists such as ranitidine (Zantac) and famotidine (Pepcid), and mast cell membrane stabilizers such as oral cromolyn sodium (Gastrocrom) [32–36]. Because of the nocturnal awakening observed in our patients, we would also suggest adding an antileukotriene such as montelukast (Singulair) or a 5-liopoxygenase inhibitor such as zileuton extended-release tablets (Zyflo CR). In patients with more severe symptoms that significantly disrupt their activities of daily living and/or sleep, we suggest the addition of budesonide (Entocort) or a short course of prednisone.

In conclusion, herein we report a cohort of patients with gastrointestinal mast cell disease separate and distinct from systemic mastocytosis and the aforementioned GI mast cell disorders. Further characterization of this possible disorder is needed in order to clearly distinguish these patients from those with IBS or systemic mastocytosis.

## Figures and Tables

**Table 1:** Summary of signs and symptoms of the subjects (*n* = 24).

Sign or Symptom	Present	Not Present	Not Available
Abdominal pain	24 (100%)	0 (0%)	0 (0%)
Early satiety/bloating	23 (95.8%)	1 (4.2%)	0 (0%)
Constipation	9 (37.5%)	15 (62.5%)	0 (0%)
Diarrhea	11 (45.8%)	13 (54.2%)	0 (0%)
Succussion splash	13 (54.2%)	5 (20.8%)	6 (25%)
Nocturnal awakening	19 (79.2%)	0 (0%)	5 (20.8%)

**Table 2:** Summary of the allergic history of the subjects (*n* = 24).

History	Positive	Negative	Not available
Immunotherapy	8 (33.3%)	16 (66.7%)	0 (0%)
Food allergy	9 (37.5%)	11 (45.8%)	4 (16.7%)
Environmental allergy	16 (66.7%)	2 (8.3%)	6 (25%)

**Table 3:** Summary of solid-phase gastric emptying time (minutes).

	Solid-phase gastric emptying time (min)
Total number of values available	14
Minimum25% percentileMedian75% percentileMaximum	10.086.5132.0338.75500.0
MeanStd. deviationStd. error	204.357162.6743.4753
Lower 95% CI* of meanUpper 95% CI* of mean	110.434298.28

*CI: confidence interval.

**Table 4:** Summary of the GI tract biopsy/CD117 staining results: number of mast cells per high power field (hpf).

# Mast cells/hpf	Stomach	Small Bowel	Colon
Total number of values available	16	22	13
Minimum	16.0	30.0	1.0
25% percentile	30.75	39.0	28.0
Median	35.5	48.5	40.0
75% percentile	43.0	70.0	47.5
Maximum	82.0	90.0	68.8333
Mean	39.25	52.9545	37.9103
Std. deviation	15.7628	17.5105	17.177
Std. error	3.94071	3.73325	4.76404
Lower 95% CI* of mean	30.8506	45.1908	27.5304
Upper 95% CI* of mean	47.6494	60.7183	48.2902

*CI: confidence interval.

**Table 5:** Summary of serum IgE levels and whole blood histamine levels.

	Total IgE IU/mL	Histamine nmol/L
Total number of values available	22	20
Minimum25% percentileMedian75% percentileMaximum	3.014.7537.090.01556.0	<300547.75776.01025.751597.0
MeanStd. deviationStd. error	213.409465.57399.2605	798.1319.25571.3876
Lower 95% CI* of meanUpper 95% CI* of mean	6.98497419.833	648.682947.518

*CI: confidence interval.

**Table 6:** Comparison between different GI mast cell diseases [20, 24].

	Cardinal symptoms	Number of mast cells/HPF	Serum markers
Mastocytic enterocolitis	Abdominal pain, diarrhea	>20	N/A
Mast cell Activation syndrome	Abdominal pain, dermatographism, flushing	17–23	Serum tryptase
Allergic mastocytic Gastroenteritis and colitis	Abdominal pain, dysmotilitysymptoms (e.g., early satiety, bloating), nocturnal awakening	>40	Histamine, IgE
